# Psychometric properties of a short version of the HIV stigma scale, adapted for children with HIV infection

**DOI:** 10.1186/1477-7525-11-195

**Published:** 2013-11-14

**Authors:** Maria Wiklander, Lise-Lott Rydström, Britt-Marie Ygge, Lars Navér, Lena Wettergren, Lars E Eriksson

**Affiliations:** 1 Department of Neurobiology, Care Sciences and Society, Karolinska Institutet, Huddinge, Sweden; 2 Department of Clinical Sciences Danderyd Hospital, Karolinska Institutet, Stockholm, Sweden; 3 Department of Pediatrics, Karolinska University Hospital, Stockholm, Sweden; 4 Department of Women’s and Children’s Health, Karolinska Institutet, Stockholm, Sweden; 5 Department of Clinical Science, Intervention and Technology, Karolinska Institutet, Stockholm, Sweden; 6 Department of Infectious Diseases, Karolinska University Hospital, Huddinge, Sweden; 7 School of Health Sciences, City University London, London, UK

**Keywords:** HIV, Stigma, Child, Adolescent, Psychometrics, HIV Stigma Scale for Children

## Abstract

**Background:**

HIV is a stigmatizing medical condition. The concept of HIV stigma is multifaceted, with personalized stigma (perceived stigmatizing consequences of others knowing of their HIV status), disclosure concerns, negative self-image, and concerns with public attitudes described as core aspects of stigma for individuals with HIV infection. There is limited research on HIV stigma in children. The aim of this study was to test a short version of the 40-item HIV Stigma Scale (HSS-40), adapted for 8–18 years old children with HIV infection living in Sweden.

**Methods:**

A Swedish version of the HSS-40 was adapted for children by an expert panel and evaluated by think aloud interviews. A preliminary short version with twelve items covering the four dimensions of stigma in the HSS-40 was tested. The psychometric evaluation included inspection of missing values, principal component analysis (PCA), internal consistency, and correlations with measures of health-related quality of life (HRQoL).

**Results:**

Fifty-eight children, representing 71% of all children with HIV infection in Sweden meeting the inclusion criteria, completed the 12-item questionnaire. Four items concerning participants’ experiences of others’ reactions to their HIV had unacceptable rates of missing values and were therefore excluded. The remaining items constituted an 8-item scale, the HIV Stigma Scale for Children (HSSC-8), measuring HIV-related disclosure concerns, negative self-image, and concerns with public attitudes. Evidence for internal validity was supported by a PCA, suggesting a three factor solution with all items loading on the same subscales as in the original HSS-40. The scale demonstrated acceptable internal consistency, with exception for the disclosure concerns subscale. Evidence for external validity was supported in correlational analyses with measures of HRQoL, where higher levels of stigma correlated with poorer HRQoL.

**Conclusion:**

The results suggest feasibility, reliability, as well as internal and external validity of the HSSC-8, an HIV stigma scale for children with HIV infection, measuring disclosure concerns, negative self-image, and concerns with public attitudes. The present study shows that different aspects of HIV stigma can be assessed among children with HIV in the age group 8–18.

## Background

About 34 million individuals are presently living with HIV worldwide [1]. The corresponding figure for Sweden is approximately 6000 individuals [2]. About 150 of these are children under the age of 19 who have an early acquired HIV infection (perinatally infected) [3]. Paediatric HIV was once a fatal disease, where most children with HIV infection died at an early age. Today, mother to child transmission of HIV is preventable in most cases if treatment is available. However, some children still acquire HIV. Reasons for that could be if the mother’s HIV infection is unknown or if treatment is unavailable. For children who are HIV infected, combined antiretroviral treatment (cART) has changed HIV from a fatal to chronic condition [4,5]. Among children with HIV infection an increasing number is reaching adulthood, with long life-expectancy.

Despite the progress in prevention and treatment of the disease, HIV-related stigma remains to impact the lives of individuals living with HIV [6-11]. The concept of stigma was defined by Erving Goffman [12] as an “attribute that is deeply discrediting” that reduces the individual “from a whole and usual person to a tainted, discounted one” (p. 3). A theoretical model suggested by Earnshaw and Chaudoir (2009) describes the differences in stigma mechanisms experienced by individuals with, respective without, HIV. For individuals with HIV infection, HIV stigma is expressed as experiences and anticipation of stigmatization from others as well as internalized stigma, while HIV stigma in HIV-uninfected individuals is expressed as prejudice, stereotypes, and discrimination [13].

Although stigma among adults with HIV infection has been investigated to some extent (e.g. [6-11]), there is limited research on HIV stigma in children and most of the existing literature does not distinguish between children who have HIV themselves and children who are affected by HIV (e.g. orphans whose parents have died from AIDS [14,15]). Since it is a relatively new phenomenon that children with HIV survive into adulthood, the consequences of stigma specific for HIV-infected children have not been much studied. Consequently, there is also limited research on which stigma mechanisms (e.g. internalized or anticipated stigma) might be influencing children with HIV infection. Having an inborn stigmatizing condition might result in different stigma experiences, than having acquired the stigmatizing condition as adult (cf. [12] pp. 32–40). However, a recent qualitative study from our research group indicates that young adults with perinatally acquired HIV experience stigma mechanisms similar to those infected with HIV as adults [16]. The consequences of HIV stigma are different for HIV-infected and HIV-affected children in a number of areas. Individuals who have HIV infection are restricted by HIV-specific laws in many countries, including Sweden [17]. Further, the associations between HIV stigma and quality of life outcomes are different for HIV-infected and HIV-affected children. For example, HIV-infected children might experience side-effects, symptoms, and life restrictions associated with their HIV infection. Interventions are needed for different reasons for HIV-infected and HIV-affected children. Interventions against HIV stigma for HIV-infected children might need to target treatment adherence and life issues including intimate relationships. In sum, as pointed out by Earnshaw and Chaudoir, the consequences of HIV stigma are different for infected and uninfected individuals [13].

Instruments to measure HIV-related stigma in adults have been developed and validated in different contexts (e.g. [18-20]). One of the scales, the widely used 40-item HIV Stigma Scale (HSS-40) by Berger et al. (2001) [6], has the advantage that it is differentiates between all three stigma mechanisms (enacted, anticipated, internalized) for HIV-infected individuals, as suggested by Earnshaw and Chaudoir [13]. Two short versions of the HSS-40 have been adapted to and used in studies with young adults with HIV [21,22]. However, to our knowledge, there are no instruments for HIV-infected children under the age of fifteen. As has been emphasized in several HIV stigma reviews and reports (e.g. [23-25]), sound instruments to measure HIV stigma are essential for the understanding of different expressions of HIV stigma, its consequences, and intervention outcomes. Therefore, the aim of the present study was to test an adapted short version of the HSS-40 for 8–18 years old children with HIV infection, living in Sweden.

## Methods

### Definition

The term “child” will be used for individuals under the age of nineteen in this article.

### Adaptation of the HIV Stigma Scale

**Figure 1:**
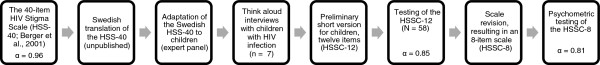
Work flow of the adaptation and testing of the HIV Stigma Scale for Children (HSSC-8).

The HSS-40 is a 40-item instrument for assessment of HIV stigma in adults with HIV infection [6]. The instrument was developed based on a wide review of the literature and it has been used in several studies in different contexts (e.g. [7,10,26]). The HSS-40 measures four dimensions of HIV stigma: 1) *personalized stigma*, perceived stigmatizing consequences of others knowing of one’s HIV status; 2) *disclosure concerns*, fear of disclosing one’s own HIV status and fear that those who know would tell others; 3) *negative self-image*, experiencing oneself as tainted and not as good as others because of one’s HIV; and 4) *concerns with public attitudes*, conceptions of what people might think about a person with HIV. Responses to 38 of 40 statements are rated on a four-point Likert scale from “strongly disagree” to “strongly agree”, where higher scores indicate higher level of stigma. The remaining two items are reversely phrased, with statements suggesting that HIV is not stigmatizing. For items describing situations they have not actually experienced (e.g.; ‘I have been hurt by how people reacted to learning I have HIV’), the respondents are instructed to imagine their reactions and respond accordingly. In addition to the summarized scores on each of the four dimension subscales, a total score can be obtained. The scale has previously been translated into Swedish using a bilingual committee of HIV/infectious disease experts, using a translation and back-translation procedure. For the present study, the language was slightly adapted to children by pediatric experts working with HIV. The adaptation process is presented in Figure 1.

Children’s understanding and interpretation of the 40 adapted items was evaluated with think aloud methodology [27], with a convenience sample of seven 11–16 years old children with HIV infection. The participants were instructed to verbalize their thoughts and reactions when completing the questionnaire. The think aloud interviews were performed by coauthor L-LR and lasted between 30 and 40 minutes.

Based on the results from the think aloud interviews and the dimensions of the HSS-40 presented by Berger et al. (2001), the expert panel selected items for a short version for children (HSSC-12; Table 1). The criteria for selection of items were: (1) high factor loadings in the original HSS-40, (2) comprehensibility, as assessed by the think aloud interviews, (3) each subscale represented by three items, which has been recommended as the minimum number of items per factor, for factor analysis [28]. All items were statements about the presence of different stigma mechanisms, where agreement indicated that the individual experienced HIV stigma. Four of the items concerned others’ reactions to the participants’ HIV. As it was not expected that all children had told others about their HIV, they were instructed in the questionnaire to imagine their responses to situations they had not actually experienced. For clarity, no reversed items were used in the scale.

**Table 1:** **Items, stigma dimensions and missing values in the HSSC-12, items retained in the final scale marked with **^**a**^**(N = 58)**

**Item**	**Dimension**	**Missing values (%)**
1. I work hard to keep my HIV a secret^**a**^	Disclosure concerns	0.0
2. Most people believe a person who has HIV is dirty^**a**^	Public attitudes	3.4
3. Having HIV makes me feel unclean^**a**^	Negative self-image	8.6
4. Most people think a person with HIV is disgusting^**a**^	Public attitudes	1.7
5. Having HIV makes me feel I'm a bad person^**a**^	Negative self-image	0.0
6. Most with HIV are rejected when others learn^**a**^	Public attitudes	5.2
7. I am very careful whom I tell that I have HIV^**a**^	Disclosure concerns	3.4
8. Having HIV in my body feels disgusting^**a**^	Negative self-image	0.0
9. I have been hurt by how people reacted to learning I have HIV	Personalized stigma	19.0
10. I worry that people who know I have HIV will tell others	Disclosure concerns	13.8
11. I have stopped socializing with some due to their reactions to my HIV	Personalized stigma	19.0
12. I have lost friends by telling them I have HIV	Personalized stigma	24.1

*Abbreviation*: HSSC-12 = HIV Stigma Scale for Children, preliminary 12-item version.

^a^Items retained in the final scale.

### Other measures

In addition to the HSSC-12, the participants completed a health-related quality of life (HRQoL) questionnaire for children with a chronic medical condition, the 37-item DISABKIDS Chronic Generic Module (DCGM-37) including six dimensions [29]. Data from three of these dimensions, Emotion, Social Inclusion, and Social Exclusion, were used for validation of the final version of the HIV stigma scale for children (see below). The Emotion subscale measures negative emotions such as anger and sadness, the Social Inclusion subscale measures acceptance from and positive relationships with others, and the Social Exclusion subscale measures feelings of being left out and being treated differently than others [29]. All scores in DCGM-37 are transformed into a 0–100 scale, where higher scores indicate better HRQoL.

### Participants

All clinics caring for 8–18 years old children with HIV infection in Sweden were asked to invite patients who matched the inclusion criteria to participate in the study. The inclusion criteria were: (1) having a perinatally or early acquired HIV infection; (2) attending HIV care in Sweden for more than five years; (3) being 8–18 years old at the time of invitation; (4) being informed about his or her HIV status; and (5) understanding and speaking Swedish.

### Procedure

Data was collected October 2011 through October 2012. The participants received written and oral information about the study, and were invited to participate by their responsible nurse or physician. Written informed consent was obtained from participants aged 15 or older. For children younger than 15 years, consent was additionally obtained from the child’s legal guardian.

The questionnaires were completed in connection with a scheduled visit at the hospital, or handed out in connection with a visit at the hospital and completed at home and sent to the research team by mail. Demographic data such as country of birth was collected by the responsible nurse. The study was approved by the Regional Ethical Review Board of Stockholm, Sweden (2011/1120-32).

### Statistical analysis

Comparisons between participants and non-participants were calculated with independent samples *t*-test for age and *χ*^2^-test for sex. Feasibility of scale items was assessed by inspection of missing values. Items with more than 10% missing values were considered unfeasible [30]. For exploration of factor structure, a principal component analysis (PCA) with varimax rotation was performed. As suggested by Field (2009) [31], a sample size of 50 individuals can be satisfactory for factor analysis, if more restrictive thresholds for interpretation of factor loading are employed. Therefore, a sample size of 50 was set as minimum for PCA and 0.722 was set as a minimum for interpretation of factor loadings [31]. Adequacy of the data for factor analysis was investigated with the Kaiser-Meyer-Olkin measure of sampling adequacy (KMO) and Bartlett’s test of sphericity [31]. Factors were extracted, using Kaiser’s criterion of retaining factors with eigenvalues of >1, and inspection of the scree plot [32]. Characteristics of the extracted factors (subscales), as well as intercorrelations between the scale and its subscales, were evaluated for increased evidence of internal validity. Internal consistency reliabilities of the scale and its subscales were assessed with Cronbach’s α. For evidence of external validity, Spearman’s ρ correlations between the final version of the HIV Stigma Scale for Children (HSSC-8; see below) and three subscales from the DCGM-37 were used. It was hypothesized that HIV stigma as measured with the HSSC-8 would be associated with poorer HRQoL, as measured with the DCGM-37 subscales Emotion, Social exclusion and Social inclusion. For all analyses, *p* <0.05 was considered statistically significant. All statistical analyses were conducted with IBM SPSS 20 (IBM Corp., Armonk, NY).

## Results

### Participants

Fifty-eight out of 82 eligible children completed the HSSC-12 questionnaire. The respondents were 27 females and 31 males, aged between 9 and 18 (mean 13.9, SD 2.5). No eight years old children were eligible in this sample. The vast majority of the participants were immigrants, born outside Europe.

The response rate was 71%. Of the 24 non-respondents, eleven children themselves or their legal guardians actively declined participation in the study. In thirteen cases, the reason for non-response was unknown. The participants did not differ from non-participants regarding age (*t*[80] 1.67, *p* 0.10) or sex (*χ*^2^[1, n = 82] 0.39, *p* 0.53)*.*

### Scale

#### Feasibility

The HSSC-12 was evaluated with respect to missing values (Table 1). The four items measuring experiences of others’ reactions to the participants’ HIV had unacceptably high rates of missing values and were excluded from further analyses. Of those items, three concerned personalized stigma (missing values 19–24%), and one concerned fear that people who knew about the HIV would tell others (disclosure concerns; missing values 14%). This means that the whole personalized stigma subscale was excluded together with one item from the disclosure concerns subscale (with two items remaining). Further assessments of validity were performed on the revised 8-item version of the scale, named the HIV Stigma Scale for Children (HSSC-8).

#### Internal structure

The sample size in the PCA was 52 (participants with complete data). Evidence for internal validity of the HSSC-8 was supported by the PCA. The data was adequate for factor analysis; the KMO value was 0.77 and Bartlett’s test of sphericity was statistically significant (χ^2^ 140.0, *p* <0.001). In the PCA, a three-factor solution was suggested, with all items loading on the same factors as in the original HSS-40 (Table 2). The three factors accounted for 72.4% of the total variance (component 1: negative self-image 27.5%; component 2: public attitudes 27.0%; and component 3: disclosure concerns 17.8%, with varimax rotation).

**Table 2:** Factor loadings based on principal component analysis with varimax rotation of the HSSC-8 (n = 52)

	**Component**		
	**1**	**2**	**3**
Having HIV makes me feel unclean	**0.866**	0.302	0.102
Having HIV makes me feel I'm a bad person	**0.816**	0.139	0.192
Having HIV in my body feels disgusting	**0.732**	0.192	-0.027
Most people think a person with HIV is disgusting	0.233	**0.833**	0.145
Most with HIV are rejected when others learn	0.105	**0.811**	0.100
Most people believe a person who has HIV is dirty	0.329	**0.794**	0.086
I am very careful whom I tell that I have HIV	-0.053	0.079	**0.873**
I work hard to keep my HIV a secret	0.269	0.167	**0.759**

*Abbreviations*: HSSC-8 = HIV Stigma Scale for Children, Component 1: Negative self-image, Component 2: Public attitudes, Component 3: Disclosure concerns.

#### Scale characteristics

The HSSC-8 consisted of three subscales, based on the results from the PCA. The subscales were disclosure concerns (2 items), negative self-image (3 items), and concerns with public attitudes (3 items). The scales were inspected for floor and ceiling effects. Forty-one percent of the participants received a maximum score on the disclosure subscale. Fifty-nine and nineteen percent respectively received minimum scores on the negative self-image and public attitudes subscales. Descriptive statistics are presented in Table 3. Due to the skewed distributions of the subscales, the relationships between the HSSC-8 and its subscales were calculated with Spearman’s ρ. The correlations between the HSSC-8 and the subscales were in the range ρ 0.59 - 0.88, while the correlations between the subscales were in the range ρ 0.23 – 0.42. The results are presented in Table 4.

**Table 3:** Descriptive statistics for the HSSC-8 and its three subscales (N = 58)

**Scale (Possible range)**	**n**	**Min- max**	**Mean scale score (SD)**	**Floor/ ceiling effects**^**a **^**(%)**	**Reliability (α)**
HSSC-8 (8–32)	52	10-30	17.44 (5.13)	0/0	0.81
Disclosure concerns (2–8)	56	2-8	6.64 (1.52)	1.8/41.1	0.55
Negative self-image (3–12)	58	3-11	4.33 (2.16)	58.6/0	0.78
Public attitudes (3–12)	53	3-12	6.51 (2.85)	18.9/5.7	0.80

*Abbreviations*: HSSC-8 = HIV Stigma Scale for Children, ^a^Percentage of responses at the minimum or maximum value of the scale.

**Table 4:** Bivariate Spearman’s ρ correlations of the HSSC-8 with subscales and the DCGM-37 subscales Emotion, Social Exclusion, and Social Inclusion (n = 49-58)

	**HSSC-8 Disclosure**	**HSSC-8 Negative self-image**	**HSSC-8 Public attitudes**	**DCGM-37 Emotion**	**DCGM-37 Social exclusion**	**DCGM-37 Social inclusion**
HSSC-8 Total	0.65^***^	0.59^***^	0.88^***^	- 0.47^**^	- 0.37^**^	- 0.38^**^
HSSC-8 Disclosure		0.23	0.32^*^	- 0.29^*^	- 0.27^*^	- 0.41^**^
HSSC-8 Negative self-image			0.42^**^	- 0.49^***^	- 0.36^**^	- 0.26
HSSC-8 Public attitudes				- 0.37^**^	- 0.28^*^	- 0.33*

*Abbreviations*: HSSC-8 = HIV Stigma Scale for Children, DCGM-37 = DISABKIDS Chronic Generic Module. ^*^ < 0.05, ^**^ < 0.01, ^***^ < 0.001.

#### Reliability

The HSSC-8 had good internal consistency (Cronbach’s α 0.81). The 3-item public attitudes and the 3-item negative self-image subscales were internally consistent (α 0.80 and 0.78, respectively). The 2-item disclosure subscale did not reach the standards for good reliability (α 0.55).

#### External validity

Evidence for external validity of the HSSC-8 was supported by correlational analyses with the DCGM-37 (Table 4). There was an inverse relationship between HIV stigma and HRQoL, with correlations between the HSSC-8 (and its subscales), and the subscales from the DCGM-37 in the range ρ 0.26 – 0.49 (Table 4).

## Discussion

In this study, a short version of the HSS-40 [6] was adapted for and tested on 8–18 years old children with HIV infection. The study resulted in an 8-item stigma scale, the HSSC-8, measuring disclosure concerns, negative self-image, and concerns with public attitudes among children with HIV infection. The scale appeared feasible, generally reliable (except for low Cronbach’s α on the 2-item disclosure concerns subscale) and had a good internal factor structure, with items loading on the same factors as in the original HSS-40. The HSSC-8 was inversely related to subscales from the DCGM-37, supporting external validity and demonstrating that HIV stigma is associated with poorer HRQoL in children. The HSSC-8 is, to our knowledge, the first stigma scale designed for children under the age of fifteen living with HIV infection.

The prevalence of HIV infection among children in Sweden is low and although 71% of all eligible children participated in the study, this resulted in a relatively small sample (n = 52) for factor analysis, which is one of the major limitations of the study. Therefore, we set a threshold of 0.722 for interpretation of factor loadings, as suggested by Field (2009) [31]. Furthermore, it has been demonstrated that valid factor solutions can be obtained with small samples, providing that the communalities are high (average above 0.7) and the factors are overdetermined (at least three or four variables in each factor) [33]. In the present study, the average of communalities was 0.723 and two of the three factors were determined by three variables. As this is the first evaluation of the scale, the factor structure is recommended to be evaluated further. The other major limitation concerned psychometric problems with the 2-item disclosure concerns subscale. Firstly, it is generally suggested that factors should have at least three items [28]. Secondly, the subscale had low reliability, which could be a result of the number of items. It’s also debated how reliability in 2-item scales should be assessed and some authors discourage from the use of Cronbach’s α in 2-item scales [34]. Therefore, it is suggested that an additional item measuring disclosure concerns is added in future studies using HSSC-8.

The original HSS-40 [6] measures four dimensions of stigma: personalized stigma, disclosure concerns, negative self-image, and concerns with public attitudes. The preliminary version of the questionnaire constructed for the present study contained twelve items (HSSC-12), covering all these dimensions. However, unfeasibility of the four items measuring the participants’ experiences of others’ reactions to their HIV, led to a reduction of the preliminary 12-item scale into the final 8-item scale, with no personalized stigma items and only two disclosure concerns items. It was common that only the families and healthcare workers knew about the participants’ HIV. The participants had been instructed to imagine their responses to situations they had not experienced, but the rates of missing values indicate that this instruction was difficult to follow. The think aloud interviews further confirmed the difficulty of these items for children who had not revealed their HIV status to people outside the family. However, personalized stigma items might be relevant and possible to complete for HIV-infected children in other contexts. In Sweden, the low prevalence of HIV might lead to low levels of expressed prejudice, stereotyping and discrimination towards people with HIV, and low awareness of the existence of children with HIV infection in Sweden. This, together with the limited disclosure of the participants’ HIV status to others, might explain the lack of recognition of personalized stigma among children in this sample. Since HIV stigma is a social concept [12,35,36] with different impact and expressions in different contexts, the relevance of personalized HIV stigma among infected children should be assessed anew in other contexts.

## Conclusions

The HSSC-8 is an 8-item short version of the HSS-40 [6], measuring HIV-related disclosure concerns, negative self-image, and concerns with public attitudes among 8–18 years old children with HIV infection. The results of the study suggest that HSSC-8 is a feasible, generally reliable, and valid instrument to assess HIV-related stigma in children with HIV infection. However, as this is the first study using the HSSC-8, further evaluation is suggested.

## Abbreviations

cART: Combined antiretroviral treatment; DCGM-37: DISABKIDS Chronic Generic Module; HRQoL: Health-related quality of life; HSS-40: HIV Stigma Scale; HSSC-12: HIV Stigma Scale for Children preliminary 12-item version; HSSC-8: HIV Stigma Scale for Children; KMO: Kaiser-Meyer-Olkin measure of sampling adequacy; PCA: Principal component analysis.

## Competing interest

The authors declare no conflict of interest.

## Authors’ contributions

LEE and L-LR conceived and designed the study, MW, LEE and L-LR conducted the analyses and, together with LW, interpreted the results. MW and L-LR drafted the manuscript. LEE, B-MY, LN, and LW contributed in the critical drafting and revising of the manuscript for important intellectual content. All authors read and approved the final manuscript.
